# Treatment outcomes for idiopathic sudden sensorineural hearing loss in dialysis patients

**DOI:** 10.1038/s41598-023-49306-8

**Published:** 2024-01-03

**Authors:** Seonju Kim, Dong Kyu Lee, Hae-rim Kim, Jung Mee Park, Soon Bae Kim, Hoon Yu

**Affiliations:** 1 Division of Nephrology, Department of Internal Medicine, College of Medicine, Asan Medical Center, University of Ulsan, Seoul, 05505 Republic of Korea; 2 Department of Otorhinolaryngology—Head and Neck Surgery, College of Medicine, Asan Medical Center, University of Ulsan, Seoul, 05505 Republic of Korea; 3 College of Natural Science, School of Statistics, University of Seoul, Seoul, 02504 Republic of Korea; 4 Department of Otorhinolaryngology—Head and Neck Surgery, College of Medicine, Gangneung Asan Hospital,, University of Ulsan, Gangneung, 25440 Republic of Korea; 5 Division of Nephrology, Department of Internal Medicine, College of Medicine, Gangneung Asan Hospital, University of Ulsan, Gangneung, 25440 Republic of Korea

**Keywords:** Kidney, Outcomes research

## Abstract

Idiopathic sudden sensorineural hearing loss (ISSNHL) is challenging for both nephrologists and otolaryngologists treating patients undergoing dialysis. This single-center, retrospective, observational study investigated the treatment outcomes of patients with ISSNHL undergoing dialysis, enrolling 700 patients (47 undergoing and 653 not undergoing dialysis) diagnosed with ISSNHL between January 2005 and December 2021 at Asan Medical Center, Republic of Korea. To balance pre-existing clinical characteristics, 1:5 propensity score matching (PSM) was performed with the patients who were not undergoing dialysis. Treatment included high-dose systemic steroid therapy or intra-tympanic steroid injections. The pure tone average of the groups was compared before and 2 weeks and 2 months after treatment. The hearing-improvement degree was evaluated using Siegel's criteria. Before PSM, age, prevalence of diabetes or hypertension, initial hearing threshold at each frequency level (0.5, 1, 2, and 4 kHz), and treatment strategies exhibited significant between-group differences. However, in the PS-matched cohort, none of the confounders showed significant between-group differences. Two months after steroid treatment, the non-dialysis patient group demonstrated significantly higher average improvement in pure tone audiometry (*P* = 0.029) and greater percentage of complete response according to Siegel's criteria. This study suggests that treatment outcomes for ISSNHL are significantly poorer for patients undergoing than for those not undergoing dialysis.

## Introduction

Idiopathic sudden sensorineural hearing loss (ISSNHL) is characterized by a sudden occurrence of SNHL with a minimum threshold drop of 30 dB within 3 days, affecting at least three consecutive frequencies on pure tone audiometry^1^. Treating SSNHL in patients undergoing dialysis poses a challenge for nephrologists owing to the unique condition of these patients and the limited clinical experience available. Currently, glucocorticoids are considered the primary treatment for SSNHL, despite the inconclusive evidence of their efficacy^2^. Glucocorticoids can be administered either systemically or via intra-tympanic (IT) injection. The latter is often used as a secondary option when systemic therapy fails to improve hearing levels, and it may be used simultaneously with systemic therapy or as the primary therapy for patients concerned of the side effects of high-dose systemic glucocorticoids, such as those with uncontrolled diabetes mellitus.

Based on a systematic review that investigated the impact of hemodialysis (HD) on hearing in patients with chronic kidney disease (CKD), while there is no definitive evidence establishing HD as the sole cause of SSNHL, a substantial body of research suggests a positive correlation^3^, implying that HD may play a significant role in the development of SSNHL. Studies have reported that the prevalence of SSNHL among patients with CKD or end-stage renal disease (ESRD) is as high as 75%, which is significantly higher than that in the general population^4–6^. Factors, such as exposure to ototoxic medications, imbalances in electrolyte and osmotic pressure, acute neuritis resulting from rapid ultrafiltration, and immunological similarities between the kidney and cochlea, are thought to contribute to hearing loss in patients with ESRD^7–9^. However, the etiology of SSNHL in patients undergoing dialysis remains unknown, as there have been limited studies and case reports published on SSNHL in patients with ESRD. Although few studies have compared the treatment outcomes of ISSNHL between the general population and patients undergoing dialysis, the available reports suggest that patients with ESRD have poorer ISSNHL treatment outcomes than those expected for the general population^2,10–13^. This difference in outcomes can be attributed to various factors, including disrupted electrolyte and acid–base balance, alterations in peri-dialysis pharmacokinetics, and uncontrolled uremia, which may negatively impact the treatment response in patients undergoing dialysis. However, to date, no studies have effectively controlled for various confounding variables to clearly delineate the distinct clinical features and treatment outcomes between patients undergoing and not undergoing dialysis. The primary objective of this study was to investigate whether there would be differences in the treatment outcomes and prognosis of ISSNHL between dialysis and non-dialysis groups, even after accounting for potential confounding variables through propensity score matching (PSM), with the ultimate aim of providing a more comprehensive perspective on the management of patients with ISSNHL undergoing dialysis.

## Results

### Baseline characteristics of the study patients before PSM

**Figure 1:**
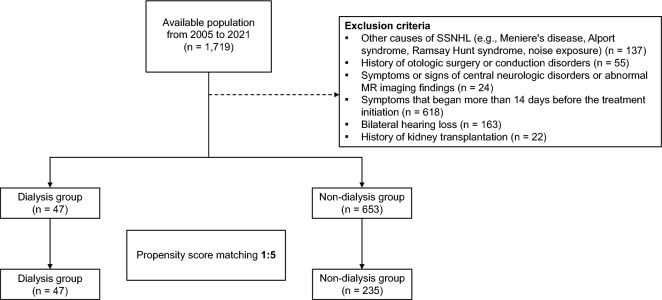
Cohort creation flow chart.

Among the 700 patients included in this study, 14.9% were receiving dialysis, while 85.1% were not. The recruitment process for the study population is shown in Fig. 1. At baseline, the patients had a mean age of 58.1 years, and 350 (50.0%) were male. The mean pure tone audiometry threshold of the affected side at the initial presentation was 67.61 ± 27.95 dB. Within the dialysis group, 39 patients (75.5%) had undergone HD and eight (24.5%) had undergone peritoneal dialysis for a median duration of 47 months (range, 1–210). In the dialysis group, the diseases predisposing patients to ESRD were as follows: diabetic nephropathy in 23 (48.9%), chronic glomerulonephritis in 5 (10.6%), hypertensive nephropathy in 3 (6.4%), polycystic kidney disease in 3 (6.4%), systemic lupus erythematosus in 2 (4.3%), chronic vasculitis in 1 (2.1%), and unknown causes in 10 (21.3%) patients. In comparison to the non-dialysis group, the dialysis group showed a significantly higher prevalence of diabetes (59.6% vs. 24.3%; *P* < 0.001) and hypertension (83.0% vs. 35.1%; *P* < 0.001), a higher mean pure tone average (PTA) threshold (83.91 ± 23.54 vs. 66.43 ± 27.89; *P* < 0.001), and a higher proportion of patients treated with IT steroid injection (87.2% vs. 63.4%; *P* < 0.001). However, the dialysis group included fewer older patients (53.57 ± 10.77 vs. 58.41 ± 12.67; *P* = 0.011; Table 1).

**Table 1:** Characteristics of patients with ISSNHL undergoing and not undergoing dialysis before and after PSM.

Characteristics	Before PSM			After PSM		
	Non-dialysis group (n = 653)	Dialysis group (n = 47)	*P*-value	Non-dialysis group (n = 235)	Dialysis group (n = 47)	*P*-value
Sex (%)						
Female	329 (50.4)	21 (44.7)	0.546	108 (46.0)	21 (44.7)	1
Male	324 (49.6)	26 (55.3)		127 (54.0)	26 (55.3)	
Age (years)	58.41 ± 12.67	53.57 ± 10.77	0.011	57.02 ± 12.61	53.57 ± 10.77	0.082
Body mass index (kg/m^2^)	24.45 ± 3.86	23.41 ± 4.03	0.078	24.12 ± 3.72	23.41 ± 4.03	0.24
Delayed time before treatment	3.36 ± 3.07	3.23 ± 3.32	0.792	3.36 ± 3.15	3.23 ± 3.32	0.808
Diabetes mellitus (%)	159 (24.3)	28 (59.6)	< 0.001	106 (45.1)	28 (59.6)	0.098
Hypertension (%)	229 (35.1)	39 (83.0)	< 0.001	165 (70.2)	39 (83.0)	0.108
Treatment strategy (%)						
Systemic steroids only	239 (36.6)	6 (12.8)	< 0.001	53 (22.6)	6 (12.8)	0.087
Systemic + IT steroids	351 (53.8)	27 (57.4)		141 (60.0)	27 (57.4)	
IT steroids only	63 (9.6)	14 (29.8)		41 (17.4)	14 (29.8)	
Initial pure tone threshold according to frequency (mean ± SD)						
500 Hz (threshold, dB)	64.72 ± 29.07	81.70 ± 27.51	< 0.001	73.79 ± 30.98	81.70 ± 27.51	0.105
1000 Hz (threshold, dB)	67.30 ± 29.97	83.40 ± 29.71	< 0.001	76.28 ± 33.13	83.40 ± 29.71	0.172
2000 Hz (threshold, dB)	65.20 ± 30.62	83.40 ± 25.54	< 0.001	75.74 ± 33.01	83.40 ± 25.54	0.134
4000 Hz (threshold, dB)	68.51 ± 30.26	89.47 ± 21.22	< 0.001	82.53 ± 29.84	89.47 ± 21.22	0.13
*Pure tone average (threshold, dB)	66.43 ± 27.89	83.91 ± 23.54	< 0.001	77.09 ± 29.73	83.91 ± 23.54	0.139

*This is the value of the unmatched data.

*dB* decibel, *ISSNHL* idiopathic sudden sensorineural hearing loss, *PSM* propensity score matching, *SD* standard deviation.

### Baseline characteristics of the study patients after PSM

**Figure 2:**
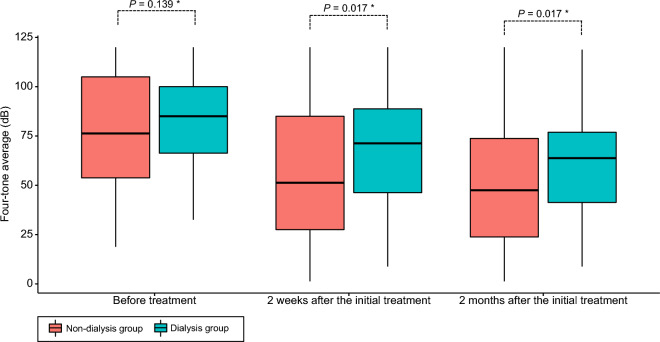
Hearing improvement according to the four-tone average (dB) 2 weeks and 2 months after treatment between the dialysis and non-dialysis groups. *Wilcoxon-test.

**Figure 3:**
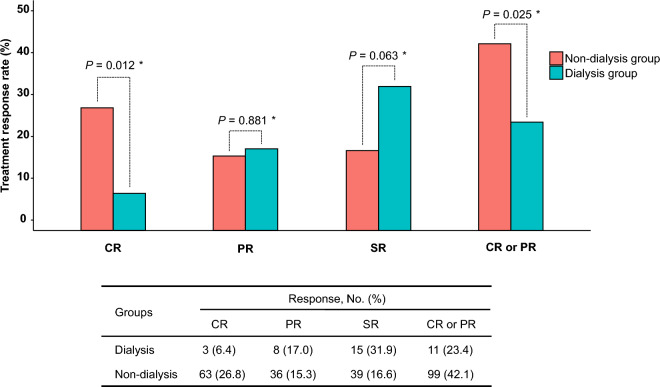
Comparison of the treatment response rate according to Siegel's criteria 2 months after treatment. Statistically significant differences were observed between the dialysis and non-dialysis groups in the CR or PR ratio. *Multivariate adjusted logistic regression.

**Figure 4:**
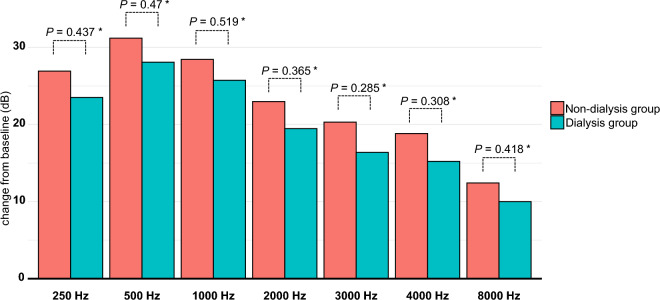
Improvement of the hearing threshold according to frequency. No statistically significant differences were observed between the dialysis and non-dialysis groups at any frequency level. *Student′s t-test.

To minimize the potential influence of confounding variables when comparing the treatment outcomes of ISSNHL between the dialysis and non-dialysis groups, we established a 1:5 PS-matched cohort. In this PS-matched cohort, there was no significant difference in baseline characteristics between the dialysis and non-dialysis groups (Table 1). Specifically, age, sex, the time interval from onset to treatment, and initial hearing levels at four frequencies were similar in both groups. At initial presentation, the mean PTA threshold of the affected side was 83.91 ± 23.54 dB in the dialysis group and 77.09 ± 29.73 dB in the non-dialysis group, with no statistically significant difference observed between the two groups (*P* = 0.139) (Table 2). After 2 weeks of steroid treatment, both groups showed significant improvement in PTA. In the dialysis group, the PTA decreased from 83.91 ± 23.54 to 68.96 ± 29.78 dB, while in the non-dialysis group, the PTA decreased from 77.09 ± 29.73 to 57.15 ± 33.56 dB; the difference in improvement was found to be statistically significant (*P* = 0.026). Similarly, a statistically significant difference in PTA improvement was observed 2 months after steroid treatment. In the dialysis group, the PTA decreased from 83.91 ± 23.54 to 62.37 ± 26.89 dB, while in the non-dialysis group, it decreased from 77.09 ± 29.73 to 51.72 ± 31.04 dB (*P* = 0.029). The improvement in PTA 2 weeks and 2 months after treatment is also depicted in Fig. 2. When evaluating the rates of hearing recovery according to Siegel’s criteria, significant differences were found in the ratio of complete response (CR) or partial response (PR) between the groups after 2 weeks of steroid treatment (dialysis group vs. non-dialysis group: 19.1% vs. 34.9%; adjusted odds ratio (AOR) = 0.34, 95% CI 0.12–0.86; *P* = 0.03) (Table 3). Similarly, significant differences in the CR or PR ratio were observed after 2 months of steroid treatment (dialysis group vs. non-dialysis group: 23.4% vs. 42.1%; AOR = 0.36, 95% CI 0.14–0.85; *P* = 0.025) (Fig. 3). No significant differences were observed between the dialysis and non-dialysis groups in the analysis of final hearing improvements according to frequency (0.25, 0.5, 1, 2, 3, 4, 6, and 8 kHz) (Fig. 4), indicating that no specific frequency significantly improved the treatment response compared to the others and suggesting that the treatment efficacy was not frequency dependent.

**Table 2:** Pure tone average according to time after treatment in the PS-matched cohort.

		Total	Dialysis group	Non-dialysis group	*P*-value
		(n = 282)	(n = 47)	(n = 235)	
Before the initial treatment					
Pure tone average (threshold, dB)	Mean ± SD	78.22 ± 28.87	83.91 ± 23.54	77.09 ± 29.73	0.139
	Median [Q1–Q3]	78.75 [55.00;103.75]	85.00 [66.25;100.00]	76.25 [53.75;105.00]	0.163
2 weeks after the initial treatment					
Pure tone average (threshold, dB)	Mean ± SD	59.12 ± 33.20	68.96 ± 29.78	57.15 ± 33.56	0.026
	Median [Q1–Q3]	55.00 [31.25;86.25]	71.25 [46.25;88.75]	51.25 [27.50;85.00]	0.017
2 months after the initial treatment					
Pure tone average (threshold, dB)	Mean ± SD	53.49 ± 30.60	62.37 ± 26.89	51.72 ± 31.04	0.029
	Median [Q1–Q3]	50.62 [27.50;73.75]	63.75 [41.25;76.88]	47.50 [23.75;73.75]	0.017

*dB* decibel, *PS* propensity score, *Q* quantile, *SD* standard deviation.

**Table 3:** Improvement in hearing according to Siegel's criteria at 2 weeks and 2 months after treatment in the PS-matched cohort.

1:5 propensity-score matched patients								
		Total (n = 282)	Dialysis group (n = 47)	Non-dialysis group (n = 235)	Crude model		Adjusted model	
					OR (95% CI)	*P*-value	OR (95% CI)	*P*-value
2-week follow up								
CR (%)		55 (19.5)	4 (8.5)	51 (21.7)	0.34 (0.10–0.88)	0.046	0.16 (0.03–0.64)	0.018
PR (%)		36 (12.8)	5 (10.6)	31 (13.2)	0.78 (0.26–1.97)	0.633	0.88 (0.27–2.40)	0.810
SR (%)		44 (15.6)	9 (19.1)	35 (14.9)	1.35 (0.57–2.94)	0.464	1.28 (0.49–3.07)	0.598
No improvement (%)		147 (52.1)	29 (61.7)	118 (50.2)	1.60 (0.85–3.08)	0.152	2.08 (0.99–4.55)	0.059
CR or PR (%)		91 (32.3)	9 (19.1)	82 (34.9)	0.44 (0.19–0.92)	0.039	0.34 (0.12–0.86)	0.03
CR, PR or SR (%)		135 (47.9)	18 (38.3)	117 (49.8)	0.63 (0.32–1.18)	0.152	0.48 (0.22–1.01)	0.059
2-month follow up								
CR (%)		66 (23.4)	3 (6.4)	63 (26.8)	0.19 (0.04–0.53)	0.006	0.15 (0.03–0.57)	0.012
PR (%)		44 (15.6)	8 (17.0)	36 (15.3)	1.13 (0.46–2.52)	0.769	1.07 (0.41–2.58)	0.881
SR (%)		54 (19.1)	15 (31.9)	39 (16.6)	2.36 (1.14–4.71)	0.017	2.15 (0.94–4.80)	0.063
No improvement (%)		118 (41.8)	21 (44.7)	97 (41.3)	1.15 (0.61–2.16)	0.666	1.41 (0.69–2.88)	0.344
CR or PR (%)		110 (39.0)	11 (23.4)	99 (42.1)	0.42 (0.20–0.84)	0.019	0.36 (0.14–0.85)	0.025
CR, PR, or SR (%)		164 (58.2)	26 (55.3)	138 (58.7)	0.87 (0.46–1.65)	0.666	0.71 (0.35–1.45)	0.344

All ORs estimated based on patients not undergoing dialysis as a reference.

Adjusted for baseline covariates including sex, age, body mass index, delayed time before treatment, diabetes mellitus, hypertension, treatment strategy, initial pure tone threshold at 500, 1000, 2000, 4000 Hz.

*CI* confidence interval, *CR* complete response, *OR* odds ratio, *PR* partial response, *PS* propensity score, *SR* slight response.

### Subgroup analysis within the PS-matched cohort

After PSM, the between-group difference in treatment strategy was found to be marginally significant (*P* = 0.087) (Table 1). Therefore, we performed an additional subgroup analysis within the PS-matched cohort to further investigate whether bias stemming from differences in treatment options between the groups existed. Regarding the achievement of CR or PR, a statistically significant difference was observed only within the treatment group administered a combination of systemic and IT steroids (OR = 0.21, CI 0.06–0.65, *P* = 0.012) (Table 4). While no significant findings were observed in the systemic steroid-only group (OR = 0.23, CI 0.03–1.45, *P* = 0.13) and IT steroid-only group (OR = 0.59, CI 0.09–3.46, *P* = 0.562), the data suggest an inferior treatment trend in the dialysis group compared to the non-dialysis group.

**Table 4:** Hearing improvement after 2 months of treatment according to treatment strategy between the dialysis and non-dialysis groups.

	Systemic steroids only		Systemic + IT steroids		IT steroids only	
	OR (95% CI)	*P*-value	OR (95% CI)	*P*-value	OR (95% CI)	*P*-value
CR	0.15 (0.01–1.17)	0.11	NA*	0.982	0.20 (0.01–2.05)	0.209
PR	1.62 (0.07–14.43)	0.695	0.61 (0.16–1.87)	0.419	3.82 (0.44–37.54)	0.225
SR	2.93 (0.04–135.78)	0.615	3.34 (1.29–8.67)	0.012	0.28 (0.01–2.71)	0.321
No improvement	3.54 (0.53–23.43)	0.176	1.09 (0.43–2.67)	0.853	4.02 (0.88–21.13)	0.082
CR or PR	0.23 (0.03–1.45)	0.13	0.21 (0.06–0.65)	0.012	0.59 (0.09–3.46)	0.562
CR, PR, or SR	0.28 (0.04–1.89)	0.176	0.92 (0.37–2.32)	0.853	0.25 (0.05–1.14)	0.082

All ORs estimated based on non-dialysis as a reference.

Adjusted for baseline covariates including sex, age, body mass index, delayed time before treatment, diabetes mellitus, hypertension, treatment strategy, initial pure tone threshold at 500; 1000; 2000; and 4000 Hz.

*Inapplicable due to the absence of CR events in the dialysis group.

*CI* confidence interval, *CR* complete response, *IT* intra-tympanic, *NA* not applicable, *OR* odds ratio, *PR* partial response, *PS* propensity score, *SR* slight response.

## Discussion

The results of our study indicate that the treatment outcomes for ISSNHL were poorer in the dialysis group than in the non-dialysis group. This study was the first to confirm a statistically significant difference in treatment outcomes between these two groups, even after adjusting for demographic factors and potential confounding variables that could influence treatment responses.

Although the precise incidence of SSNHL in patients undergoing dialysis remains unknown, Charlene et al. reported a 1.57 times higher incidence of SSNHL in patients with CKD than in controls without CKD^14^. However, limited number of reports on SSNHL in patients undergoing dialysis are available, and the relationship between dialysis and SSNHL remains unclear. Glucocorticoids have traditionally been the mainstay treatment option for ISSNHL, and current studies have suggested that the efficacy and safety of IT steroid injections are comparable to those of systemic steroid treatment^2,15–19^. Therefore, IT steroid injection is emerging as an alternative therapy for patients with systemic conditions, such as diabetes, hypertension, or CKD, which may pose challenges for the administration of systemic steroids. In our study, a higher proportion of patients undergoing dialysis received IT steroid injections compared to patients not undergoing dialysis, as patients undergoing dialysis were more likely to have underlying medical conditions. Owing to the between-group disparity in baseline characteristics, PSM was used to adjust for treatment strategies (Table 1), and neither systemic nor IT steroid treatment was associated with severe adverse effects.

Several published studies have suggested that the rate of CR or PR in the treatment of SSNHL in the general population ranges from 60 to 73%^2,10–12^. However, there is conflicting evidence regarding whether the treatment outcomes of SSNHL are poorer in patients undergoing dialysis than in patients not undergoing dialysis. Kang et al. reported a 36.4% rate of CR or PR to treatment for SSNHL in patients undergoing dialysis 2 months after steroid treatment. These results indicate that the treatment outcomes for these patients are inferior to those observed for the general population^2,11,13^. On the other hand, some studies have suggested that HD is not associated with a poor prognosis of treatment for SSNHL. Wang et al.^19^ reported on 32 patients undergoing HD derived from case studies and found that 16 (50.1%) had achieved complete or partial recovery, while nine (28.1%) had not recovered. However, in their cases, the initial hearing threshold was relatively lower and the age group was considerably younger compared to those in other studies, which likely contributed to their favorable outcomes. In a similar study conducted by Yamamoto et al.^20^, no statistically significant differences were observed in the pretreatment hearing level and recovery of the affected ear between the HD and non-HD groups (*P* = 0.12). However, a limitation of this study was the inclusion of a higher number of patients with diabetes compared to those included in the control group, which could have potentially acted as a confounding variable^21^. Furthermore, their study had a relatively smaller sample size, consisting of 23 patients undergoing dialysis and 101 patients not undergoing dialysis, compared to our study.

Hence, previous research did not consider the impact of underlying diseases, initial hearing threshold, duration of treatment delay, or differences in initial treatment methods, all of which could potentially influence treatment outcomes^22–24^. To address these concerns, our study used PSM to adjust for confounding variables. Moreover, a retrospective review of medical records spanning 15 years allowed for a relatively larger sample size compared to those in previous studies. Consequently, our findings revealed that 2 months after steroid treatment, 23.4% (11 out of 47) of patients in the dialysis group had achieved CR or PR, which was significantly lower than the 42.1% (99 of 235) in the non-dialysis group.

Although the exact mechanism underlying the development of SSNHL remains unknown, the kidney and cochlea exhibit numerous structural similarities. Both the stria vascularis of the cochlea and the glomerulus are epithelial tissues closely associated with the vascular system. Furthermore, the presence of a sodium–potassium-ATPase pump in the kidney and a carbonic-anhydrase enzyme in the cochlea have been implicated in maintaining body fluid homeostasis^25,26^. Moreover, the inner ear solely relies on the labyrinthine artery for its blood supply, which renders it susceptible to ischemic events because of its delicate vasculature^27^. Various factors, including uremia, ototoxic medication, electrolyte imbalances, and HD treatment, have been associated with hearing disorders in patients with kidney failure^7,28^. These factors suggest a shared impact of medication on these organs and strongly support the existence of a connection between hearing disorders and CKD.

In our study, we found that dialysis was associated with a poorer prognosis of treatment for SSNHL. In the PS-matched analysis, the treatment outcomes were significantly poorer in the dialysis group than in the non-dialysis group, and these findings were consistent when evaluating the average PTA values. These results indicate that dialysis itself may have an impact on the prognosis of treatment for ISSNHL.

There are several explanations for the inferior treatment outcomes for SSNHL in patients undergoing dialysis compared to patients not undergoing dialysis, although the precise mechanism remains undetermined. Firstly, patients undergoing dialysis often have multiple comorbidities, such as diabetes and hypertension, which are known contributors to the development of SSNHL^29,30^. These medical conditions can also pose challenges and reduce the efficacy of hearing treatment. Secondly, patients undergoing dialysis have a higher incidence of vascular calcification, which can lead to impaired blood flow to the inner ear and potentially contribute to SSNHL^31,32^. Additionally, calcification can pose challenges when administering medications through the blood vessels, potentially reducing treatment efficacy. Furthermore, patients undergoing dialysis may have altered pharmacokinetics and pharmacodynamics, affecting the absorption, distribution, metabolism, and elimination of drugs used in the treatment of sudden hearing loss. This can result in lower drug concentrations or altered drug effects, leading to a suboptimal therapeutic response^33,34^. However, in our study, a relatively higher proportion of patients undergoing dialysis received IT steroid treatment because of underlying medical conditions. Therefore, we conducted a subgroup analysis within the PS-matched cohort to specifically investigate whether there were significant differences in treatment outcomes based on the chosen treatment strategy, particularly between the group that received IT steroid treatment, which is free from the influence of systemic pharmacodynamics, and the group that received systemic steroid treatment. The subgroup analysis revealed significant differences only among patients who received a combination of systemic and IT steroids. Nevertheless, acknowledge the limitations of this subgroup analysis is crucial. The small sample size of the dialysis patient group had a substantial impact on the statistical power, and the possibility of administering both systemic and IT steroid treatments to patients with initially high hearing thresholds may have negatively affected the perceived prognosis. Despite these limitations, the subgroup analysis consistently indicated a trend of poorer treatment outcomes in patients undergoing dialysis than in patients not undergoing dialysis across all three treatment strategies. The reason for their poorer treatment response compared to the non-dialysis group is believed to be related to irreversible inner ear damage in the dialysis patient group, likely arising from unresolved issues such as uremia, osmotic changes resulting from dialysis, or factors such as acute neuritis caused by ultrafiltration during dialysis^8,9^. These multiple factors likely interacted in a complex manner, contributing to the observed outcomes. Larger prospective studies are warranted to substantiate these findings.

As the results of our study suggest that dialysis may have an impact on the outcomes of ISSNHL, it may be necessary to consider more intensive and prompt initiation of treatment for patients undergoing dialysis. Nevertheless, our study has several limitations. Firstly, this was a retrospective study conducted at a single center, which may have limited the generalizability of the findings. Secondly, as Asan Medical Center is a tertiary medical care facility, many patients in our study were referred from local clinics after initial treatment failure, resulting in potential selection bias, as we excluded patients with inconsistent treatment protocols. Despite these limitations, our study sample was larger than those in previous studies and used PSM to control for confounding variables to minimize bias. The clinical implications of our findings for the understanding and management of patients with ISSNHL undergoing dialysis are significant. Nevertheless, future prospective studies with larger populations are warranted to validate our findings.

Although SSNHL poses a significant complication affecting the quality of life of patients undergoing dialysis, the limited clinical experience and research in this area make it difficult for nephrologists to determine the appropriate management strategies. While our study was not prospective, it included a relatively larger number of patients compared to previous studies and yielded reliable results after adjusting for confounding variables. In our analysis, we provided first confirmation of a statistically significant difference in the treatment outcomes for ISSNHL between dialysis and non-dialysis groups. This finding suggests that dialysis may serve as a poor prognostic factor in the treatment of ISSNHL. Consequently, both nephrologists and otolaryngologists must be aware of these unfavorable outcomes when managing ISSNHL in patients undergoing dialysis. Efforts toward early diagnosis and the prompt implementation of tailored treatment strategies upon diagnosis are crucial for improving the outcomes of ISSNHL in patients undergoing dialysis.

## Methods

### Data sources and study population

We conducted a retrospective cohort study at Asan Medical Center, a 2700-bed academic tertiary referral hospital in Seoul, Republic of Korea, to compare the treatment response of patients undergoing dialysis with that of patients not undergoing dialysis. The medical records of 1719 patients diagnosed with ISSNHL between January 2005 and December 2021 were evaluated. ISSNHL was diagnosed using pure tone audiometry, following the criteria outlined in the American Academy of Otolaryngology—Head and Neck Surgery practice guideline. These criteria primarily include: (1) sudden onset of SNHL within 72 h and (2) audiometric confirmation of 30-dB hearing loss for at least three consecutive frequencies. We thoroughly reviewed the electronic medical records of all 1719 patients and excluded 1,019 patients who met the following exclusion criteria: (1) other causes of SNHL (such as Meniere’s disease or Alport syndrome); (2) a history of otologic surgery or conduction disorder; (3) symptoms or signs of central neurologic disorders or abnormal brain magnetic resonance imaging findings; (4) symptoms that started > 14 d before initiating treatment; (5) bilateral hearing loss; and (6) a history of kidney transplantation, as it is suggestive of non-idiopathic hearing loss (Fig. 1). Finally, a total of 700 patients were enrolled, including 47 patients undergoing dialysis and 653 patients not undergoing dialysis. The demographics, audiometric examinations, treatment details, and dialysis records for all included patients were reviewed.

### Treatment protocol

All enrolled patients received either systemic steroid therapy or IT steroid injections. The standard treatment for SSNHL involved administering oral methylprednisolone (48 mg) for nine s, followed by a 5-d weaning period (32 mg for 2 d, 16 mg for 2 d, and 8 mg for 1 d, for adults weighing 60 kg). An IT steroid injection was administered to patients who had uncontrolled diabetes mellitus and declined systemic steroid medication or to those who did not achieve more than partial recovery according to Siegel's criteria after 2 weeks of treatment. For 2 consecutive weeks, patients received twice-weekly IT steroid injections with a concentration of 5 mg dexamethasone. The IT steroid injection was administered with the patient in a supine position with the head tilted toward the contralateral ear. An anterosuperior puncture was made in the tympanic membrane area using a 1 mL syringe equipped with a 25-gauge spinal needle. We injected a 0.4–0.5 mL dexamethasone solution to fill the posterior tympanic area. Additional treatments, such as hyperbaric oxygen therapy, prostaglandin E therapy, or vitamins, were rarely used as adjunctive therapies.

### Treatment outcomes

To assess the changes in hearing level after the treatment, auditory evaluations were conducted before and 2 weeks and 2 months after treatment. Auditory function was measured with pure tone audiometry, and the mean hearing levels were calculated as the average of the hearing thresholds at 500; 1000; 2000; and 4000 Hz (referred to as either the pure tone average or the four-tone average). The treatment response was categorized based on Siegel’s criteria^35^, which included the following classifications: (1) CR, indicating a final hearing threshold > 25 dB; (2) PR, indicating a gain of > 15 dB with a final hearing threshold between 25 and 45 dB; (3) slight response, indicating a gain of > 15 dB with a final hearing threshold > 45 dB; and (4) no improvement, signifying a gain of < 15 dB or a final hearing threshold > 75 dB. Patients who achieved CR or PR according to Siegel’s criteria were considered to have achieved auditory recovery. Additionally, at the 2-week and 2-month follow ups, the hearing outcomes were compared as categorical measures of improvement in PTA.

### PSM

Among the 700 patients included in the study, 47 were undergoing dialysis while 653 were not. We used PSM to identify patients with similar baseline characteristics, as there were notable differences in baseline characteristics between the groups (Table 1). Propensity scores were calculated using eight variables, age, sex, presence of diabetes, presence of hypertension, body mass index, duration of treatment delay, treatment strategy, and initial hearing thresholds at frequencies of 0.5, 1, 2, and 4 kHz, with a ratio of 1:5 for patients undergoing dialysis versus patients not undergoing dialysis. Through this matching process, we obtained a subset of 282 patients who did not differ significantly in any of the measured confounding variables.

### Statistical analysis

Descriptive statistics are used to characterize the baseline characteristics of the study population. Continuous variables are presented as means ± standard deviations, while categorical variables are presented as percentages and absolute numbers. Student’s t-test and Mann–Whitney U tests were used to compare continuous variables between the two groups, whereas the chi-squared and Fisher’s exact tests were used to assess categorical variables. Within-group comparisons of improvement were assessed using a Wilcoxon-test for both the dialysis and non-dialysis groups. A sample size calculation indicated that 44 patients should be enrolled in each group to detect a 20% difference in the rate of hearing recovery between the groups, with a statistical power of 80% at an alpha level of 5%. Propensity scores were calculated for each individual and were then used to match individuals from the dialysis and non-dialysis groups. The 1:5 ratio signifies that for every patient in the treatment group, five from the control group are matched based on their propensity scores. This approach was chosen to ensure group balance, minimize bias, and maximize statistical power. We also performed a multivariate adjusted logistic regression model analysis. The multivariate model was adjusted for the same variables that were included in the PS calculation. Before creating a final adjusted model, models sequentially adjusted based on a priori considerations for baseline covariates were also evaluated (Table 3). Subgroup analyses were undertaken to assess potential treatment-related effect modifications. We stratified each group based on treatment to account for the possible influence of the treatment (Table 4). Subgroup analyses were performed using multivariate adjusted logistic regression methods. Two-sided *P* values of < 0.05 were considered significant. All statistical analyses and visualizations were conducted using R version 4.1.1 (The R Foundation, Vienna, Austria, www.R-project.org).

### Statement of eth ics

This study was conducted in accordance with the Declaration of Helsinki and was approved by the Institutional Review Board (IRB) of Asan Medical Center (Approval No.: S2022-1042-0001). The IRB of Asan Medical Center waived the requirement for informed consent because the data analyses were performed retrospectively using anonymized data derived from electronic medical records. All methods were performed in accordance with the relevant guidelines and regulations.

## Data Availability

Owing to the privacy of those who participated in the study, the data underlying this publication cannot be disclosed publicly. The data will be shared upon reasonable request to the corresponding author.

## Abbreviations

AOR: Adjusted odds ratio
CI: Confidence interval
CKD: Chronic kidney disease
CR: Complete response
dB: Decibels
ESRD: End-stage renal disease
HD: Hemodialysis
ISSNHL: Idiopathic sudden sensorineural hearing loss
IT: Intra-tympanic
OR: Odds ratio
P: *p* Value
PR: Partial response
PS: Propensity score
PSM: Propensity score matching
PTA: Pure tone average
SD: Standard deviation
SR: Slight response
